# Exploring Growth-Stage Variations in Home Use of Positioning and Mobility Assistive Technology for Children with GMFCS IV Cerebral Palsy: Parental Insights and Challenges

**DOI:** 10.3390/bioengineering12030241

**Published:** 2025-02-26

**Authors:** Hsin-Yi Kathy Cheng, Shun-Yin Hu, Yan-Ying Ju, Yu-Chun Yu

**Affiliations:** 1 Graduate Institute of Early Intervention, College of Medicine, Chang Gung University, Taoyuan 333, Taiwan; 2 Department of Physical Medicine and Rehabilitation, Chang Gung Memorial Hospital, Taoyuan 333, Taiwan; 3 Department of Adapted Physical Education, National Taiwan Sport University, Taoyuan 333, Taiwan; yanju@ntsu.edu.tw; 4 Taoyuan Municipal Taoyuan Special School, No. 10, Deshou Street, Taoyuan District, Taoyuan 330, Taiwan; ot.spring.yu@gmail.com

**Keywords:** assistive technology, disability, cerebral palsy, mobility support, postural control, parental perspectives, developmental stages

## Abstract

This study examines how the use of postural and mobility devices evolves in home environments for children with GMFCS IV cerebral palsy, focusing on parents’ perspectives on benefits, outcomes, and challenges. As children grow, changes in muscle strength, motor function, and daily activity demands necessitate adjustments in assistive devices to maintain mobility and postural support. Data from 10 parents, collected through descriptive statistics and qualitative interviews, covered device types, usage patterns, and family impacts across developmental stages from preschool to adulthood. Device needs shift significantly with growth, transitioning from early gait trainers and postural support devices to advanced mobility devices, such as power wheelchairs, which become essential in adulthood. Parents reported positive outcomes, including improved emotional well-being, social participation, and independent mobility, alongside reduced caregiving burdens. However, challenges persist, including financial constraints, frequent device replacements, and limited training for users and caregivers. These insights highlight the need for more adaptable device designs and enhanced family-centered support programs to better assist caregivers in managing device transitions. This study addresses a gap by exploring the real-world outcomes of home-based device use, providing data and parental insights to inform device design, clinical practices, and family-centered support programs. Future research should focus on enhancing device functionality, customization, and accessibility to improve quality of life and promote greater independence for individuals with cerebral palsy.

## 1. Introduction

Cerebral palsy (CP) is a lifelong motor disorder affecting movement, posture, and muscle coordination, particularly in children classified at levels III to V of the Gross Motor Function Classification System (GMFCS). Due to motor impairments, these children face significant challenges in daily activities. One of the most critical issues is limited mobility and improper posture, which not only restrict movement but also lead to skeletal deformities, muscle stiffness, respiratory difficulties, feeding and digestive issues, and pressure ulcers [1,2,3,4,5]. These complications further reduce functional independence, making assistive devices essential for improving care and quality of life [6].

Assistive devices play a crucial role for children with GMFCS IV CP, offering benefits that extend beyond basic mobility support [7,8]. In home settings, mobility aids, such as manual and power wheelchairs, are essential for independent movement, while some children may also use gait trainers for supported walking within limited distances [9,10]. These devices help children perform essential daily activities (e.g., moving between rooms) while also promoting independent mobility. Wheelchairs provide necessary support for children who cannot walk functionally, while gait trainers may be used for therapeutic walking exercises to improve overall health and muscle development.

Beyond mobility aids, positioning devices, such as adaptive seating systems and standing frames, are equally important for maintaining proper body alignment and preventing deformities [11]. For children who require full postural support, adaptive seating systems align the spine and pelvis, reduce joint contracture risk, promote better respiratory function, and facilitate feeding. Likewise, standing frames allow children to experience weight-bearing postures, improving bone density, circulation, and muscle strength.

Unlike children with lower GMFCS levels, those with GMFCS IV CP typically rely on assistive devices for postural support and functional independence [12]. However, the goals of assistive device use evolve with age and development, particularly focusing on maintaining posture, facilitating interaction, and optimizing mobility [13]. In early childhood, devices such as adaptive seating, standing frames, and supported walkers promote motor development, enabling environmental exploration and play while preventing skeletal deformities and muscle contractures [14]. Additionally, adaptive seating ensures proper posture during feeding and daily activities, reducing respiratory issues and enhancing comfort. As children grow and reach school age, assistive devices facilitate participation in daily life beyond just health maintenance. While independent ambulation remains unlikely, manual and power wheelchairs become primary mobility aids, while positioning aids, such as specialized cushions and customized seating, prevent pressure ulcers and ensure comfort during prolonged sitting. Maintaining stable posture and optimized mobility significantly impacts education, social engagement, and quality of life.

In adolescence, assistive devices focus on long-term mobility and comfort, supporting independence and reducing caregiver burden [15]. Advanced wheelchairs with positioning systems enhance mobility and prevent complications from prolonged immobility. Additionally, transfer aids facilitate safe movement between seating and bed [16], minimizing injury risk for both children and caregivers. As mobility needs evolve, maintaining proper support and comfort becomes essential for sustaining functional independence and preventing further decline in motor function.

Although assistive device use in schools is well studied, home use remains relatively underexplored [17,18,19]. Without professional supervision, parents must learn to select and use devices independently. Beyond this challenge, high costs and limited subsidies—mainly focused on schools and medical institutions—further strain families [7,20]. Frequent adjustments or replacements due to children’s growth add to the financial burden. Parent discussions on device selection and reuse highlight the urgent need for better subsidies and professional guidance to enhance home care quality.

However, limited research explores home-based assistive device use from parents’ perspectives. This study examines the patterns of assistive device use across developmental stages for children with GMFCS IV cerebral palsy in home settings. It also evaluates the functional benefits observed by parents, with a focus on how device needs, usability, and perceived outcomes change over time.

## 2. Materials and Methods

**Figure 1:**
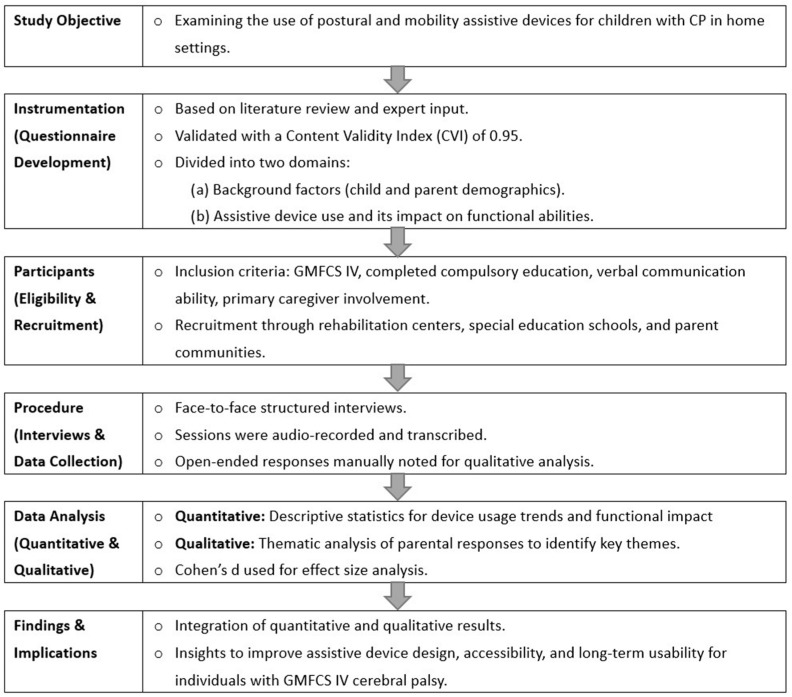
Research flowchart.

This study examines the current use of positioning and mobility-related assistive devices for children with GMFCS IV cerebral palsy in home settings, with a retrospective review across different age groups. Structured interviews were conducted with parents using a self-developed interview guide, which served as the framework for questions on assistive device usage and benefits. At the end of the interview, parents were given the opportunity to provide additional feedback (Figure 1).

### 2.1. Instrumentation

The semi-structured interview questionnaire was developed based on a literature review summarizing the use of positioning and mobility-related assistive devices for children with cerebral palsy in home settings and their impact on daily functional abilities. The positioning and mobility devices discussed in this study are categorized according to functional needs, aligning with the World Health Organization’s emphasis on mobility devices as tools to enhance personal mobility, as well as the Assistive Technology Act’s broader focus on assistive technologies that improve functional abilities in individuals with disabilities [21,22]. Based on this classification, mobility-related assistive devices include walkers, gait trainers, manual wheelchairs, power wheelchairs, and strollers, while positioning-related assistive devices include positioning chairs and standing frames.

The structured interview questionnaire was divided into two domains. The first domain focused on background factors, including parental age, gender, education level, academic background, and caregiving duration, along with child-related information, such as ICF classification, disability level, and educational background. The second domain examined assistive device usage, categorizing devices by four developmental stages: preschool, elementary school, middle/high school, and college or above. This section also assessed the impact of assistive device use on functional performance, including respiratory function, feeding and eating, digestive function, sleep quality, concentration and emotional improvement, joint deformity prevention, upper limb function, mobility, participation, and social adaptation. These impacts were measured using a 5-point Likert scale (1–5 points), with 0 points assigned to non-applicable items. Additionally, open-ended questions allowed parents to share additional insights, needs, or recommendations. The internal consistency of this domain was assessed using Cronbach’s alpha, yielding a reliability coefficient of 0.88, which indicates strong internal consistency.

To ensure content validity, the questionnaire was reviewed and refined by five experts in early intervention, assistive technology, and rehabilitation, achieving a Content Validity Index (CVI) of 0.95. The second domain also included open-ended prompts, allowing parents to elaborate on their experiences with assistive devices. A pilot test with two eligible parents was conducted to refine unclear or problematic questions based on their feedback.

### 2.2. Participants

This study recruited parents of children with cerebral palsy classified at GMFCS level IV. The inclusion criteria were designed to ensure data consistency, relevance, and the reliability of parental responses regarding assistive device use. The study required that the child had completed 12 years of compulsory education and was under 30 years old to ensure participants had experienced key developmental transitions while minimizing generational differences in technology access and caregiving practices. Additionally, the child needed to have verbal communication abilities and cognitive function above the level of mild intellectual disability to allow for meaningful caregiver insights based on the child’s self-reported experiences.

Parents were required to have no communication difficulties and be able to understand the interview questions to ensure clarity and accuracy in their responses. Furthermore, only primary caregivers who spent over 70% of their time at home caring for the child were included to ensure that responses reflected firsthand, long-term experience with assistive device use in home settings.

### 2.3. Procedure

Due to specific inclusion criteria, participants were recruited through multiple channels. The research team sought eligible parents via rehabilitation centers, special education schools, and cerebral palsy parent communities using a word-of-mouth approach. The study’s objectives were explained to potential participants, and parents who met the criteria were invited to participate. During the two-month recruitment period, 10 eligible parents agreed to participate. Interviews were conducted face-to-face by a designated interviewer, with each session lasting approximately 60 min. Sessions were audio-recorded, and quantitative data were documented directly on the questionnaire forms. Open-ended responses were either manually noted in detail or transcribed into text for further analysis. The qualitative data were analyzed using a thematic analysis approach, where transcripts were systematically reviewed to identify recurring patterns and key themes related to assistive device usage, benefits, and challenges. Coding and categorization of themes were conducted in a structured manner to ensure consistency, with careful cross-checking to enhance reliability. This method allowed for a structured interpretation of parental perspectives while maintaining the richness of individual experiences.

### 2.4. Data Analysis

This study employed a mixed-methods approach, integrating quantitative and qualitative analysis to comprehensively examine the use of positioning and mobility-related assistive devices in home settings for children with GMFCS IV cerebral palsy and their impact on daily functional performance. The analysis covered demographic information about parents and children, assistive device usage trends, perceived benefits of device use, and insights from parental open-ended responses.

Quantitative data were analyzed using descriptive statistics, summarizing demographic characteristics such as parental age and education level, as well as patterns of assistive device usage across different developmental stages. The impact of assistive device use on children’s daily functional performance was assessed through the Likert scale, examining aspects such as physiological, psychological, motor, and participation-related functions. The study also identified trends in assistive device usage across preschool, elementary school, middle/high school, and college or above stages. To ensure accuracy and completeness, all data entries were double-checked by the research team. Additionally, Cohen’s d effect size was used to evaluate the impact of different assistive devices on specific functional aspects.

Qualitative data were derived from open-ended parental responses, which were transcribed verbatim and anonymized to ensure participant privacy. The research team conducted multiple readings of the transcripts and applied open coding to identify key concepts and emerging themes. Through thematic analysis, four core themes were identified: assistive device needs, key assistive devices, impact on functional performance, and challenges or unmet needs in device usage.

To provide a comprehensive perspective, findings from the qualitative analysis were integrated with the quantitative results. By combining statistical analysis and thematic analysis, this study aimed to illustrate assistive device usage trends across different age groups, assess the functional impact of assistive devices, and uncover parents’ subjective experiences and unmet needs. These findings offer valuable insights into the future design of assistive devices and the development of educational support programs to enhance their usability and effectiveness.

## 3. Results

### 3.1. Parent and Child Background Information

A total of 10 parents participated in the study, all of whom were female and aged between 51 and 60 years. All were stay-at-home mothers, with 90% spending more than 16 h per day present with their child, including sleep time. During these hours, caregiving primarily involved providing intermittent assistance with transfers, self-care, meal preparation, and mobility support as needed rather than continuous hands-on care. Detailed information is provided in Table 1.

Regarding the children’s background, all 10 children held a government-issued Disability Certificate, which qualifies them for financial assistance and access to welfare services, including subsidies for assistive devices and healthcare support. Among them, four children were classified solely under ICF Category 7 (Neuromusculoskeletal and Movement-Related Functions), while another four had multiple disabilities, classified under ICF Category 7 combined with Category 1 (Mental Functions). The remaining two children had multiple disabilities, classified under ICF Category 7 combined with Category 3 (Sensory Functions and Pain). Additionally, 80% of the children had completed college-level education. Further details are presented in Table 2.

### 3.2. Usage of Positioning and Mobility Assistive Devices

**Figure 2:**
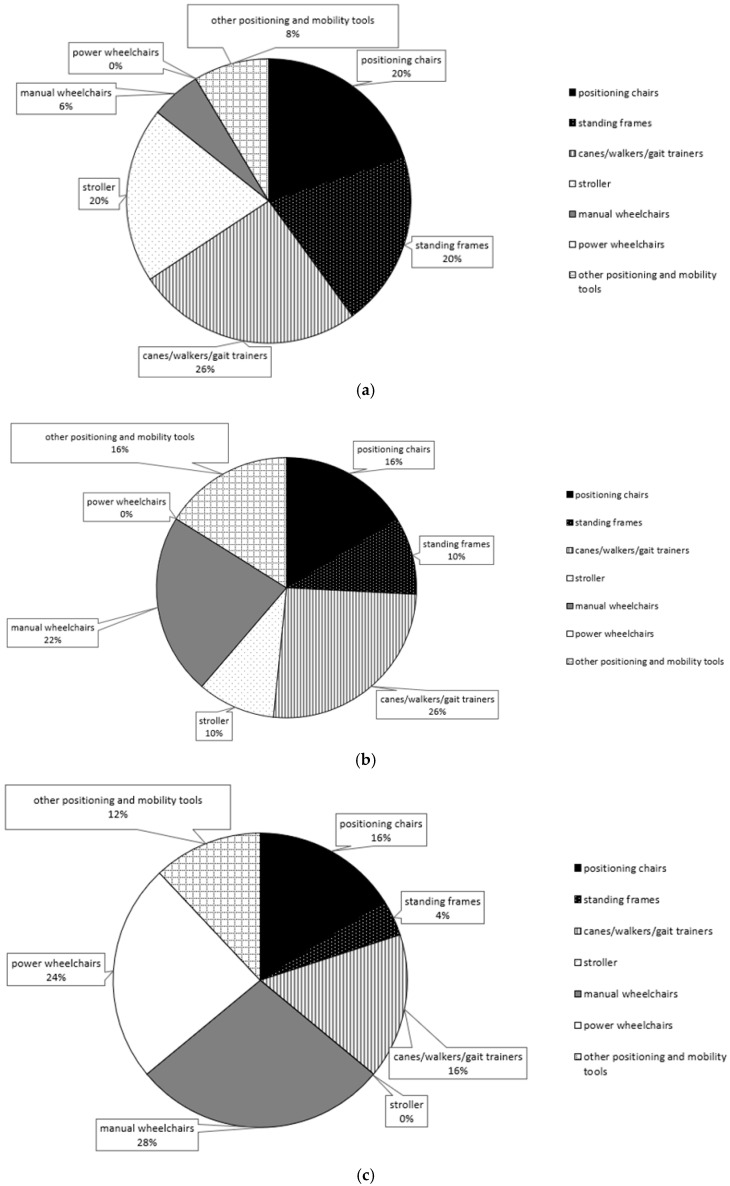

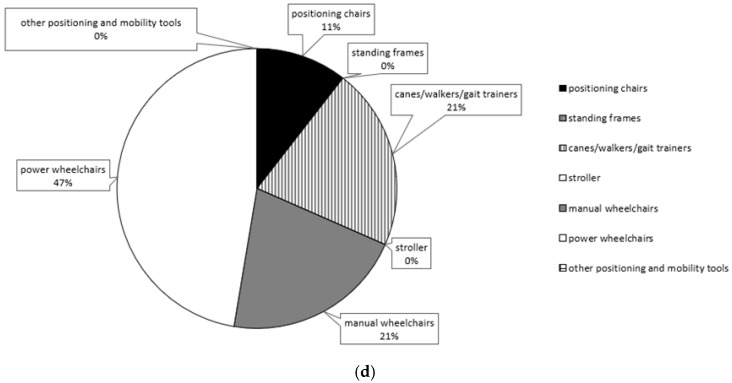
Home assistive device usage across four developmental stages: (**a**) preschool, (**b**) elementary school, (**c**) secondary school, and (**d**) postsecondary education.

This section presents the types and frequency of assistive device usage across four developmental stages: preschool, elementary school, middle/high school, and college or above. The distribution of assistive device usage at each stage is also illustrated using pie charts (Figure 2).

The assistive device categories included in the analysis are “positioning chairs”, “standing frames”, “canes/walkers/gait trainers”, “strollers”, “manual wheelchairs”, “power wheelchairs”, and “other positioning and mobility devices”.

#### 3.2.1. Preschool Stage

Statistical data indicate that the most frequently used assistive devices at this stage were “canes/walkers/gait trainers” (9 instances), followed by “positioning chairs” (7 instances), “standing frames” (7 instances), and “strollers” (7 instances). Additionally, parents reported three instances of self-made positioning and mobility assistive devices. Manual wheelchairs were used in two instances, whereas power wheelchairs were not used at all (Figure 2a).

#### 3.2.2. Elementary School Stage

During this stage, “canes/walkers/gait trainers” (eight instances) remained the most frequently used assistive devices, followed by “manual wheelchairs” (seven instances). “Positioning chairs” and “other positioning and mobility devices” were each used five times, followed by “standing frames” (three instances) and “strollers” (three instances). Power wheelchairs were not used at all during this stage (Figure 2b).

#### 3.2.3. Middle/High School Stage

At this stage, “manual wheelchairs” (seven instances) became the most frequently used assistive devices, followed by “power wheelchairs” (six instances). “Canes/walkers/gait trainers” and “positioning chairs” were each used four times, while “other positioning and mobility devices” were used three times. “Standing frames” were used once, and “strollers” were not used at all (Figure 2c).

#### 3.2.4. College and Above Stage

At this stage, “power wheelchairs” (nine instances) became the most frequently used assistive devices, followed by “manual wheelchairs” (four instances) and “canes/walkers/gait trainers” (four instances). “Positioning chairs” were used twice, while “standing frames”, “strollers”, and “other positioning and mobility devices” were not used at all (Figure 2d).

### 3.3. Impact of Assistive Device Use on Children’s Functional Performance

**Figure 3:**
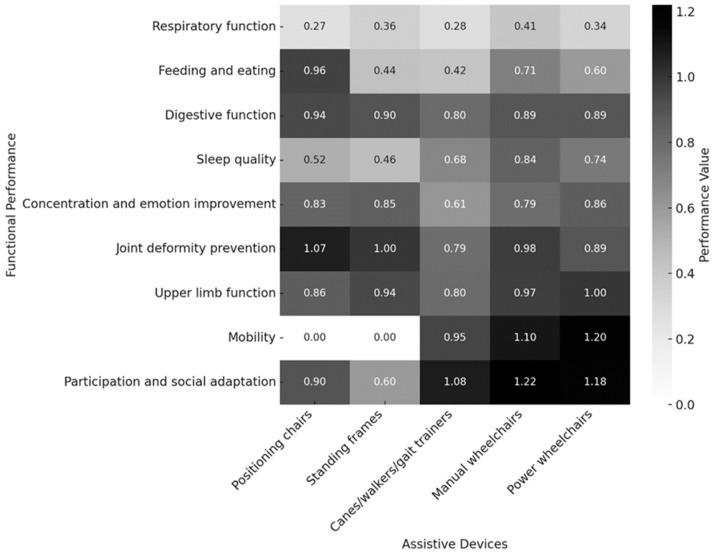
Heatmap of perceived benefits of assistive devices on functional performance.

The perceived benefits of different assistive devices on various aspects of functional performance—including respiratory function, feeding and eating, digestive function, sleep quality, attention and emotional behavior, joint deformity prevention, upper limb function, mobility, participation in daily life, and social adaptation—are illustrated in the heatmap below (Figure 3), based on parental perspectives. Since strollers were primarily used for transitions and mobility assistance, they were excluded from this analysis.

According to the data analysis, the effectiveness of assistive devices varies significantly across functional areas. For feeding and eating, as well as joint deformity prevention, positioning chairs demonstrated the greatest impact. The heatmap indicates that positioning chairs received the highest color intensity in these two categories, emphasizing their superior effectiveness in posture support and joint protection.

Similarly, standing frames exhibited a comparable impact to positioning chairs in concentration and emotion improvement, as well as joint deformity prevention, highlighting their stabilizing effect on posture control. The heatmap consistently reflects a deep color distribution for standing frames in these functions, reinforcing their role in maintaining postural stability.

To further examine these differences, an effect size analysis was conducted for various aspects of functional performance. In the effect size matrix, each numerical value represents the effect size between two assistive devices for a given function. A positive value indicates that the assistive device in the row outperforms the one in the column, while a negative value indicates the opposite. A value greater than 0.5 denotes a statistically significant difference.

The following section presents the effect size matrix for the four functional areas where assistive devices demonstrated significant differences. Matrices for other functional aspects are not displayed, as they did not exhibit statistically significant differences in effect size.

#### 3.3.1. Cohen’s D Effect Size Matrix for Feeding and Eating

The analysis revealed significant differences in assistive device effectiveness for feeding and eating. Positioning chairs had the highest score (0.96), demonstrating superior support. Manual (0.71) and power wheelchairs (0.60) provided moderate assistance, while standing frames (0.44) and canes/walkers (0.42) were the least effective.

**Figure 4:**
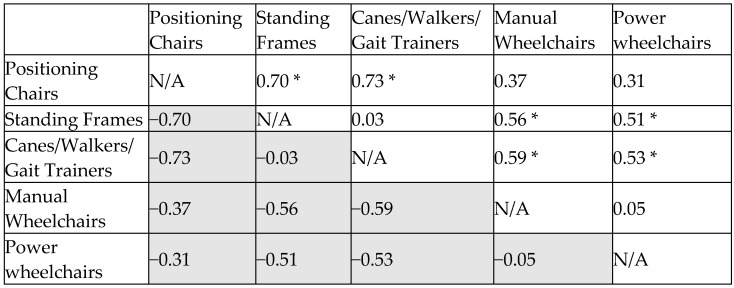
Effect size matrix for feeding and eating. *: statistically significant difference.

Cohen’s d analysis confirmed that positioning chairs significantly outperformed standing frames (0.70) and canes/walkers (0.73). Differences between positioning chairs and wheelchairs were smaller (0.31–0.37) but still favored positioning chairs. Manual and power wheelchairs had negligible differences (0.05), as did standing frames and canes/walkers (0.03) (Figure 4).

Overall, positioning chairs are the best choice for feeding support, particularly for users requiring prolonged postural stability. Wheelchairs provide secondary support, while standing frames and canes/walkers are less effective. To enhance feeding function, positioning chairs should be prioritized, with wheelchairs as needed for mobility.

#### 3.3.2. Cohen’s D Effect Size Matrix for Joint Deformity Prevention

The assessment of joint deformity prevention revealed significant differences in assistive device effectiveness. According to the heatmap, positioning chairs had the highest score (1.07), followed by standing frames (1.00), both demonstrating superior effectiveness. Manual wheelchairs (0.98) and power wheelchairs (0.89) provided moderate support, while canes/walkers/gait trainers (0.79) were the least effective.

**Figure 5:**
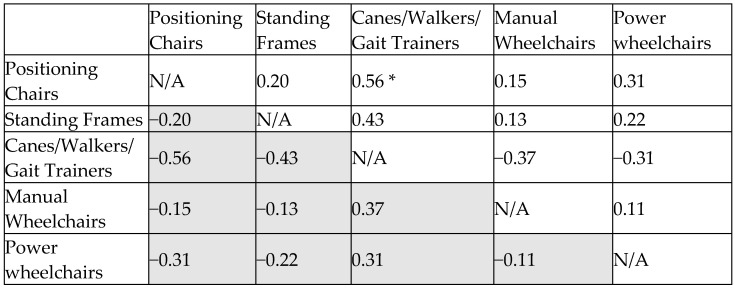
Effect size matrix for joint deformity prevention. *: statistically significant difference.

Cohen’s d analysis further quantified these differences. The effect size between positioning chairs and standing frames (0.20) showed minimal differences, whereas positioning chairs significantly outperformed canes/walkers (0.56). Differences between positioning chairs and wheelchairs were smaller (0.15–0.31), suggesting only a slight advantage. Similarly, standing frames outperformed canes/walkers (0.43) and provided slightly better joint support than wheelchairs (0.13–0.22). Manual and power wheelchairs had minimal differences (0.11) in effectiveness (Figure 5).

Overall, positioning chairs are the best option for long-term postural support and joint correction, with standing frames as a strong alternative, especially for partial weight-bearing support. Wheelchairs offer versatility, while canes/walkers are less suitable for joint deformity prevention. For optimal support, positioning chairs and standing frames should be prioritized, with wheelchairs as complementary tools based on individual needs.

#### 3.3.3. Cohen’s D Effect Size Matrix for Mobility

The assessment of mobility function showed that manual (1.10) and power wheelchairs (1.20) performed the best, demonstrating superior mobility support. In comparison, canes/walkers/gait trainers (0.95) were moderately effective but still ranked below wheelchairs. Positioning chairs and standing frames (0.00) contributed little to mobility.

**Figure 6:**
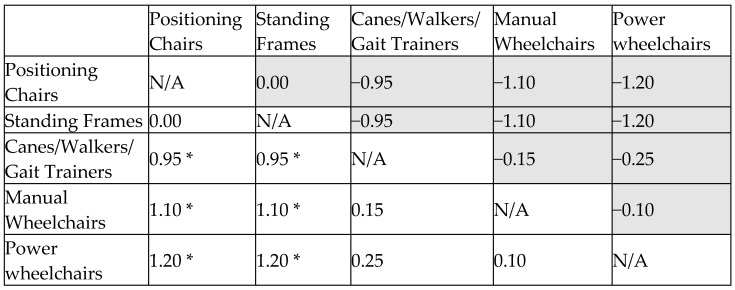
Effect size matrix for mobility. *: statistically significant difference.

Cohen’s d analysis confirmed these findings. The effect size between manual and power wheelchairs (−0.10) indicated no significant difference, allowing users to choose based on individual needs. Comparisons between wheelchairs and canes/walkers (0.15–0.25) showed a slight advantage for wheelchairs, though not statistically significant. However, when compared to positioning chairs and standing frames, the effect sizes exceeded 1.10 and 1.20, confirming wheelchairs’ significant mobility advantage (Figure 6).

Overall, manual and power wheelchairs are the best choices for users requiring high mobility, with minimal differences between them. Canes/walkers, though less effective, remain viable for short-distance mobility or for those retaining some independent movement. Power wheelchairs are recommended for long-distance mobility, while manual wheelchairs suit shorter-distance needs. Canes/walkers assist with partial independent mobility, whereas positioning chairs and standing frames should focus on postural support rather than mobility improvement.

#### 3.3.4. Cohen’s D Effect Size Matrix for Participation and Social Adaptation

The assessment showed manual (1.22) and power wheelchairs (1.18) were most effective, while canes/walkers (1.08) performed slightly lower but remained useful. Positioning chairs (0.90) and standing frames (0.60) were least effective.

**Figure 7:**
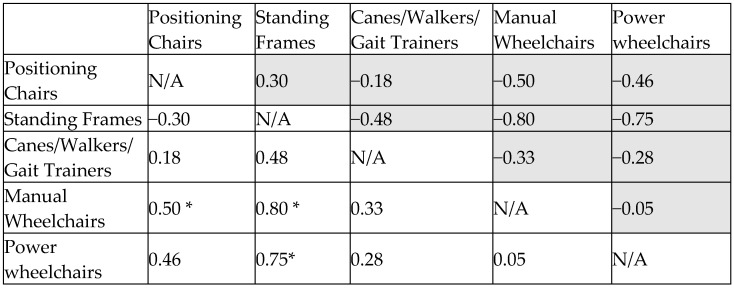
Effect size matrix for participation and social adaptation. *: statistically significant difference.

Cohen’s d analysis confirmed no significant difference between manual and power wheelchairs (−0.05). Wheelchairs outperformed canes/walkers (0.28–0.33), though the difference was modest. Larger gaps appeared when wheelchairs were compared to positioning chairs and standing frames (0.46–0.80), confirming wheelchairs’ clear advantage (Figure 7).

Overall, wheelchairs are the best choice for social participation, with power wheelchairs suited for prolonged engagement and manual wheelchairs for ease of use. Canes/walkers remain an option for lower mobility needs, while positioning chairs and standing frames should focus on postural support rather than social participation.

#### 3.3.5. Summary of Cohen’s D Effect Size Findings

This study highlights the distinct functional differences among assistive devices. Positioning chairs are most effective for postural correction and feeding support, while standing frames provide stable postural control. Wheelchairs, particularly power and manual models, are the best tools for enhancing life participation.

To optimize assistive device use, design improvements should align with specific functional needs. Enhancing feeding support in standing frames and redesigning canes and walkers for better social participation support could further expand their effectiveness.

### 3.4. Qualitative Analysis of Parents’ Responses

This section summarizes parents’ open-ended responses across four themes: assistive device needs, essential devices, functional impact, and challenges/unmet needs. A thematic analysis approach was used to identify these themes, where responses were systematically reviewed, coded, and grouped into meaningful categories. Initial coding was conducted to extract key concepts from the responses, which were then iteratively refined into broader themes based on recurring patterns.

Responses were categorized as positive, neutral, or negative, with mixed responses considered in the final summary (Table 3, Table 4, Table 5 and Table 6). To ensure consistency, coding was cross-checked, and discrepancies were resolved through discussion. This structured process allowed for a comprehensive and systematic interpretation of parental perspectives on assistive device use.

#### 3.4.1. Assistive Device Needs

Parents reported high demand for manual wheelchairs in elementary and middle/high school, supporting mobility at school and home. Power wheelchair demand increased in high school and adulthood, enhancing independence and mobility range. However, limited home space and the financial burden from frequent replacements posed major challenges (Table 3).

#### 3.4.2. Essential Assistive Devices

Positioning devices were critical for postural stability, emotional well-being, and family participation. Mobility aids supported standing and movement, preventing trunk disuse and limb deformities. Some parents raised safety concerns about power wheelchairs, particularly for younger children (Table 4).

#### 3.4.3. Impact on Functional Performance

Assistive devices improved school participation, reduced fatigue, and stabilized emotions. Power wheelchairs were essential for higher education, employment, and social interaction in adulthood. However, standing frames became less effective in middle/high school due to space constraints and time limitations (Table 5).

#### 3.4.4. Challenges and Unmet Needs

Professional guidance was crucial for optimal device selection and improving quality of life. However, financial burdens and limited professional support, especially during COVID-19, restricted accessibility. Parents emphasized the need for safer, more stable, and multifunctional assistive devices (Table 6).

## 4. Discussion

### 4.1. Growth Stages and Changing Assistive Device Needs

This study integrates quantitative data and parental feedback to examine how assistive device needs evolve across developmental stages in children with cerebral palsy. Device selection must consider both current needs and future adaptability [23]. Findings show stage-specific shifts in device types, functions, and usage, aligning with existing research on evolving assistive needs (Figure 2). These transitions can be understood through theoretical models such as the Human Activity Assistive Technology (HAAT) model, which emphasizes the interaction between the individual, the assistive device, and the environment across different life stages [24,25]. Additionally, the Life Course Theory (LCT) provides a framework for understanding how assistive needs change over time in response to developmental, social, and environmental influences [26]. Incorporating these perspectives supports the observed patterns of device transition and highlights the importance of continuous reassessment to ensure optimal assistive technology use.

In early childhood, canes/walkers/gait trainers (nine instances) were most used for mobility development, while positioning chairs, standing frames, and strollers (seven instances each) supported posture and daily movement. Manual (two instances) and power wheelchairs (zero instances) were rarely used. Parents valued positioning devices for stability but cited limited options and high costs [27].

As children grew, manual wheelchair use (seven instances) increased, supporting long-distance mobility and participation. Canes/walkers/gait trainers (eight instances) remained relevant but declined slightly as gait improved. Postural support devices (five instances each) were still used but less frequently, while standing frames and strollers (three instances each) saw further reductions. Parents reported financial strain and environmental constraints as key concerns.

By adolescence, mobility became the primary focus, with manual (seven instances) and power wheelchairs (six instances) essential for daily activities and social engagement. Canes/walkers and positioning chairs (four instances each) declined, while standing frames (one instance) and strollers (zero instances) were nearly phased out. Parents emphasized the need for more adaptable devices to address skeletal and muscle tone challenges.

In adulthood, power wheelchairs (nine instances) dominated, enabling independence and long-distance mobility. Manual wheelchairs and canes/walkers (four instances each) remained useful in specific settings, while positioning chairs (two instances) and other devices were no longer used. Parents highlighted wheelchairs’ critical role in quality of life and social participation but stressed the need for safety training and financial support.

### 4.2. Functional Impact of Assistive Devices

The heatmap in Figure 3 illustrates the perceived effectiveness of different assistive devices across various functional aspects, such as mobility, posture, joint deformity prevention, respiratory function, feeding, and participation.

Positioning chairs and standing frames were most effective for postural stability, joint deformity prevention, and feeding, as they help maintain proper spinal alignment and weight distribution. Parents reported that these devices improved sitting stability, digestion, and respiratory function, though standing frames were less utilized in adolescence and adulthood.

Manual and power wheelchairs had the greatest impact on mobility, social participation, and independence, especially in older age groups. The heatmap highlights a transition from gait trainers in early childhood to manual wheelchairs in school-age years and power wheelchairs in adulthood, reflecting changing mobility needs. However, power wheelchairs showed lower effectiveness for posture and joint support, indicating a need for supplementary positioning interventions.

For feeding and eating, positioning chairs were preferred over wheelchairs, reinforcing their role in postural control during fine motor activities. Canes, walkers, and gait trainers provided early mobility benefits but had limited long-term effectiveness, suggesting that they serve more as transitional aids rather than primary mobility solutions.

Interestingly, most assistive devices showed limited impact on sleep quality and concentration, indicating that these aspects may be influenced by external factors such as environment and overall health. This suggests that while assistive devices support mobility and posture, complementary interventions may be needed for broader well-being.

### 4.3. Overall Trends and Parental Feedback

This study shows that assistive device needs evolve with growth, shifting from postural support in early childhood to mobility assistance in adulthood. Parents emphasized early intervention and positioning devices in preschool years, while power wheelchairs became essential in adulthood. However, in the context of this study, the focus remained on the home use of assistive devices. Since power mobility is primarily utilized for outdoor activities, such as school and community participation, its presence in home settings was naturally lower.

Parents widely recognized assistive devices’ positive impact on functional performance, particularly in stabilizing posture and enhancing daily participation, aligning with previous research [28,29]. Positioning devices ensure postural stability, while wheelchairs significantly improve life participation [30]. Many parents of children with GMFCS IV cerebral palsy still hoped to develop some level of walking ability, leading to the early use of gait trainers. Modern gait trainers, equipped with supportive components, allow children with adequate cognition and stepping motivation to engage in assisted walking. Due to cultural expectations in the study region, parents often prioritized walking before transitioning to wheelchair-based mobility.

Findings also highlight how home environments influence device selection, underscoring the need for policy support. Assistive device choices are limited by household space and financial constraints [8,31]. These challenges are not unique to our study region but have also been reported in countries with varying healthcare and funding systems. Research has shown that limited home space and financial concerns frequently restrict assistive device use worldwide, even in regions with strong public funding [7,31]. By examining these challenges in different contexts, future research can help identify strategies to improve assistive device accessibility across diverse settings. Additionally, policies should provide financial assistance to reduce costs and encourage the development of multifunctional designs better suited for home use, minimizing the need for frequent replacements [32].

Additionally, educational programs for parents should provide training on device selection, operation, and maintenance, maximizing effectiveness and usability. Strengthening assistive device policies would improve access, enhance family well-being, and support a better quality of life for children with cerebral palsy.

### 4.4. Study Limitation and Contribution

This study examines assistive device experiences among parents of children with cerebral palsy (CP), a hard-to-reach population with limited prior research. Despite a small sample size, it provides valuable data on assistive device usage across different developmental stages, addressing a key research gap. Although not statistically significant, the descriptive statistics reveal meaningful trends, offering practical insights for device development, policy design, and clinical practice. The primary caregivers’ specialized experiences provide unique, irreplaceable perspectives on real-world assistive device use. Findings also serve as a foundation for future research, highlighting patterns in assistive device needs and informing larger-scale studies. While small-sample research may not be generalizable, it remains a valuable reference for professionals working with CP populations.

This study’s mixed-methods approach integrates qualitative and quantitative data, providing a comprehensive analysis of parental needs and challenges. Despite sample size limitations, it offers expert-driven insights into assistive device trends, contributing to policy and clinical practice. To enhance the generalizability of findings, future studies should expand recruitment by collaborating with additional rehabilitation centers, assistive technology providers, and caregiver support networks to reach a more diverse participant pool. Considering the traditional developmental approach observed in our study, longitudinal research should investigate the potential benefits of introducing power mobility devices earlier, aligning with modern recommendations for “On-Time Mobility”. Additionally, follow-up studies could further explore the evolving assistive device needs of individuals with CP over time, strengthening the applicability of these findings. As this study was conducted in a single geographical region, future research should explore how healthcare policies and cultural differences influence assistive device adoption. Future research with larger samples is needed to enhance external validity and broader applicability.

### 4.5. Broad Scope Impact

The findings of this study extend beyond individual experiences, offering important insights for the fields of assistive technology and rehabilitation engineering. By highlighting how assistive device needs evolve with growth, the study underscores the necessity for adaptive, user-centered designs that can accommodate changing physical and environmental demands over time. Additionally, the results emphasize the broader role of assistive devices in promoting autonomy and fostering inclusive environments, encouraging the development of innovative technologies, improving device customization or accessibility, and informing future research and policy recommendations to enhance long-term support systems.

These findings support the need for periodic reassessments to ensure assistive devices remain appropriate as users transition through life stages. Clinically, rehabilitation professionals can integrate routine evaluations into care plans, while policymakers can improve funding policies to cover device modifications and replacements. Expanding caregiver and provider training on assistive device selection and usability can further enhance long-term effectiveness. These insights provide a foundation for advancing interdisciplinary approaches that support lifelong participation for individuals with disabilities.

## 5. Conclusions

This study highlights the evolving assistive device needs of children with GMFCS IV cerebral palsy, emphasizing the critical role of timely adaptation to maintain mobility, independence, and quality of life. By integrating parental insights with quantitative data, the findings provide valuable guidance for clinicians, policymakers, and device manufacturers to enhance assistive technology design, accessibility, and long-term effectiveness. Addressing a key research gap in home-based device use, this work underscores the need for continuous reassessment, improved funding strategies, and better caregiver training to ensure sustainable support. Further large-scale studies are essential to validate these findings and refine strategies that enhance long-term well-being and social inclusion for individuals with cerebral palsy.

## Figures and Tables

**Table 1:** Demographic information of parents (N = 10).

Background Variable	Category	Count	%
Gender	Female	10	100
	Male	0	0
Age	<51	0	0
	≥51–60	10	100
	>60	0	0
Education Level	High School or Associate Degree	7	70
	Bachelor’s Degree	2	20
	Graduate School	1	10
Field of Study	Education and Related Studies	0	0
	Healthcare-related Field	0	0
	Others	10	100
Job Type	Stay-at-home Parent	10	100
	Others	0	0
Hours of Care Per Day	<8 h	0	0
	8–16 h	1	10
	≥16 h	9	90

**Table 2:** Demographic information of cerebral palsy children (N = 10).

Background Variable	Category	Count	%
Holds a Disability Certificate	Yes	10	100
	No	0	0
ICF Category	Category 7	4	40
	7 & 1	4	40
	7 & 3	2	20
Level of Disability	GMFCS I-III	0	0
	GMFCS IV	10	100
	GMFCS V	0	0
Education Level	High School or Associate Degree	2	20
	Bachelor’s Degree	8	80
	Graduate School	0	0

**Table 3:** Summary of the usage and needs of assistive devices at home as perceived by parents.

Positive	Neutral	Negative	No Comments
N = 3	N = 4	N = 2	N = 1
Manual wheelchairs were highly demanded during elementary and middle/high school stages, as parents considered them essential for mobility support at school and home.	As children grow older, their assistive devices gradually change, requiring regular assessment and adjustments.	Multiple assistive devices are needed, but limited household space makes storage difficult. This issue becomes particularly prominent during middle and high school stages when larger storage space is required.	
The demand for power wheelchairs increased during high school and adulthood, helping expand children’s social circles and enhance their independence.		Frequent replacement of assistive devices is necessary as children’s body sizes change, posing a significant financial burden on families with limited economic resources.	
Positioning assistive devices were widely used in the preschool stage, providing stable seating and safety support, making them highly relied upon by parents.			

N: Number of responses.

**Table 4:** Summary of key assistive devices in different aspects at home as perceived by parents.

Positive	Neutral	Negative	No Comments
N = 5	N = 1	N = 5	N = 0
After using positioning assistive devices, children do not need to exert much effort to maintain their posture, leading to improved emotional well-being and greater participation in family life.	I’ve heard that positioning assistive devices can improve emotional stability and focus, but I believe it varies from person to person. My child doesn’t like being fixed in one position.	Some parents have concerns about the safety and usability of power wheelchairs, especially when their children are younger.	
Mobility assistive devices provide significant support for standing and movement while also preventing trunk disuse and limb deformities.			
Parents generally agree that power wheelchairs enhance children’s social skills and help them adapt to various terrains and daily needs.			

N: Number of responses.

**Table 5:** Summary of the impact of assistive devices on daily life at home as perceived by parents.

Positive	Neutral	Negative	No Comments
N = 4	N = 4	N = 1	N = 1
Power wheelchairs provide crucial support for education, employment, and social interactions in adulthood, helping children achieve independence.	Parents believe that the effectiveness of assistive devices at different stages may vary depending on their child’s growth and living environment.	Interviews indicate that parents perceive a reduced role of standing frames during the middle and high school stages, mainly due to limited space and time constraints.	
Both manual and power wheelchairs significantly enhance school participation and reduce fatigue.			
Positioning assistive devices improve sitting stability, which in turn benefits emotional well-being and digestive function.			

N: Number of responses.

**Table 6:** Summary of challenges and unmet needs in the use of assistive devices at home as perceived by parents.

Positive	Neutral	Negative	No Comments
N = 3	N = 5	N = 2	N = 1
Collaborating with professionals to select appropriate assistive devices significantly improves quality of life.	Parents commonly report that as their children grow, assistive device usage requires continuous adjustments, but they see this as a natural process.	Many parents reported that during the pandemic, interviews and medical consultations were limited, resulting in insufficient guidance on assistive device selection and usage.	
		Some families, due to financial constraints, were unable to purchase higher-need assistive devices, causing inconvenience in daily life.	

N: Number of responses.

## Data Availability

Data will be made available on request.

## References

[1] Jonsson U., Eek M.N., Sunnerhagen K.S., Himmelmann K. (2021). Health Conditions in Adults With Cerebral Palsy: The Association With CP Subtype and Severity of Impairments. Front. Neurol..

[2] Miller F., Bachrach S., Lennon N., O’neil M.E. (2020). Cerebral Palsy.

[3] Redstone F. (2004). The effects of seating position on the respiratory patterns of preschoolers with cerebral palsy. Int. J. Rehabil. Res..

[4] Cheng H.Y.K., Yu Y.C., Wong A.M.K., Tsai Y.S., Ju Y.Y. (2015). Effects of an eight-week whole body vibration on lower extremity muscle tone and function in children with cerebral palsy. Res. Dev. Disabil..

[5] Stasolla F. (2022). A Clinical Guide to Cerebral Palsy.

[6] Hutson J., Stommes P., Wickboldt T., Tierney S.C. (2023). Suitability of quality of life outcome measures for children with severe cerebral palsy receiving postural care interventions: A scoping review. Assist. Technol..

[7] Moen R.D., Østensjø S. (2024). Understanding the use and benefits of assistive devices among young children with cerebral palsy and their families in Norway: A cross-sectional population-based registry study. Disabil. Rehabil. Assist. Technol..

[8] Ostensjo S., Carlberg E.B., Vollestad N.K. (2005). The use and impact of assistive devices and other environmental modifications on everyday activities and care in young children with cerebral palsy. Disabil. Rehabil..

[9] Jones M.A. (2020). Wheeled mobility options and indications for children and youth with cerebral palsy. Cerebral Palsy.

[10] Saleh M., Almasri N.A., Abu-Dahab S.M.N. (2023). Determinants of functional mobility in children with cerebral palsy in three different environments: A registry-based study. Physiother. Theory Pract..

[11] Angsupaisal M., Maathuis C.G.B., Hadders-Algra M. (2015). Adaptive seating systems in children with severe cerebral palsy across International Classification of Functioning, Disability and Health for Children and Youth version domains: A systematic review. Dev. Med. Child Neurol..

[12] Paleg G.S., Williams S.A., Livingstone R.W. (2024). Supported Standing and Supported Stepping Devices for Children with Non-Ambulant Cerebral Palsy: An Interdependence and F-Words Focus. Int. J. Environ. Res. Public Health.

[13] Long T.M., Perry D.F., Rahlin M. (2016). Assistive Technology, with emphasis on positioning and mobility equipment. Physical Therapy for Children with Cerebral Palsy.

[14] Ryan S.E., Campbell K.A., Rigby P.J., Fishbein-Germon B., Hubley D., Chan B. (2009). The Impact of Adaptive Seating Devices on the Lives of Young Children with Cerebral Palsy and Their Families. Arch. Phys. Med. Rehabil..

[15] Sobus K.L., Karkos J.B. (2009). Rehabilitation Care and Management for the Individual with Cerebral Palsy, Ages 13 Through Early Adulthood. Crit. Rev. Phys. Rehabil. Med..

[16] Jadhav A., Bhamre H., Jain T., Dhatrak T.G.P. (2023). Mechanisms and analysis of patient transfer assistive devices: A systematic review. AIP Conf. Proc..

[17] Alves A.C.J., Matsukura T.S. (2011). Perception of students with cerebral palsy about the use of assistive technology resources in mainstream schools. Rev. Bras. De Educ. Espec..

[18] Karlsson P., Johnston C., Barker K. (2018). Influences on students’ assistive technology use at school: The views of classroom teachers, allied health professionals, students with cerebral palsy and their parents. Disabil. Rehabil. Assist. Technol..

[19] Huang I.C., Sugden D., Beveridge S. (2009). Assistive devices and cerebral palsy: The use of assistive devices at school by children with cerebral palsy. Child Care Health Dev..

[20] Cheng H.Y.K., Yu Y.C., Ju Y.Y., Wang Y.C., Wang C.M. (2017). Assistive Devices in Special Education: Current Application Process and Problems. J. Disabil. Res..

[21] Assistive Technology Act of 1998, Public Law 105-394, 112 Stat 3627 (1998), as amended by Public Law 117-263, enacted December 23, 2022.

[22] WHO (2011). Joint Position Paper on the Provision of Mobility Devices in Less-Resourced Settings: A Step Towards Implementation of the Convention on the Rights of Persons with Disabilities (CRPD) Related to Personal Mobility.

[23] Bolton M., Donohoe M. (2020). Ambulatory assistive devices for children and youth with cerebral palsy. Cerebral Palsy.

[24] Alvarez L., Cook A., Polgar J. (2022). Assistive technology. Rehabilitation Engineering.

[25] Ramirez A.R.G., Saturno C.E., Conte M.J., da Silva J.F., Farhat M., Garcez F.D.M.G.G., Piucco E.C., Gunel M.K. (2016). Assistive and Adaptive Technology in Cerebral Palsy. Cerebral Palsy: Current Steps.

[26] Wang R.H., Kenyon L.K. (2022). Rehabilitation engineering across the lifespan. Rehabilitation Engineering.

[27] UNICEF (2015). Assistive Technology for Children with Disabilities: Creating Opportunities for Education, Inclusion and Participation.

[28] Mensah-Gourmel J., Thépot M., Gorter J.W., Bourgain M., Kandalaft C., Chatelin A., Letellier G., Brochard S., Pons C. (2023). Assistive Products and Technology to Facilitate Activities and Participation for Children with Disabilities. Int. J. Environ. Res. Public Health.

[29] Ryan S.E. (2012). An overview of systematic reviews of adaptive seating interventions for children with cerebral palsy: Where do we go from here?. Disabil. Rehabil. Assist. Technol..

[30] Noten S., Pettersson K., Czuba T., Cloodt E., Casey J., Rodby-Bousquet E. (2024). Probability of independent walking and wheeled mobility in individuals with cerebral palsy. Dev. Med. Child Neurol..

[31] Huang I.C., Sugden D., Beveridge S. (2009). Assistive devices and cerebral palsy: Factors influencing the use of assistive devices at home by children with cerebral palsy. Child Care Health Dev..

[32] Sullivan M., Lewis M. (2000). Assistive Technology for the Very Young: Creating Responsive Environments. Infants Young Child..

